# Does air-breathing meet metabolic demands of the juvenile snakehead, *Channa argus*, in multiple conditions

**DOI:** 10.1242/bio.024448

**Published:** 2017-04-10

**Authors:** Yongli Li, Xiao Lv, Jing Zhou, Chenchen Shi, Ting Duan, Yiping Luo

**Affiliations:** 1 Key Laboratory of Freshwater Fish Reproduction and Development, Ministry of Education, School of Life Sciences, Southwest University, Chongqing 400715, China; 2 Department of Clinical Medicine, Chongqing Medical and Pharmaceutical College, Chongqing 401331, China

**Keywords:** Oxygen consumption, Fish, Metabolic demand, Air-breathing

## Abstract

The objective of this study was to examine how the respiratory metabolism of the snakehead *Channa argus* changed when it shifted from breathing water to breathing air, and how increased metabolic demands caused by temperature, feeding, and exhaustive exercise affect its survival in air. The results demonstrated that the oxygen consumption rate (MO_2_) of the snakehead was lower for aerial respiration than aquatic respiration by 12.1, 24.5 and 20.4% at 20, 25, and 30°C, respectively. Survival time was significantly shortened with increasing temperature and was negatively correlated with the resting MO_2_ in air (MO_2Air_). No obvious feeding metabolic response was observed in the snakeheads fed at 1% and 3% body mass levels while breathing air. The maximum MO_2Air_ of the snakehead after exhaustive exercise was significantly higher than the resting MO_2Air_ of the control group. The results suggest that the snakehead could survive out of water by breathing air for varying lengths of time, depending on ambient temperature and metabolic demand. Additionally, some degree of metabolic depression occurs in the snakehead when breathing air. The metabolic demand associated with exercise in the snakehead, but not that associated with feeding, can be supported by its capacity for breathing air to some extent.

## Abstract (precis)

**Summary:** The snakehead could survive out of water by breathing air for varying lengths of time, depending on ambient temperature and metabolic demand.

## INTRODUCTION

The snakehead *Channa argus* is a species of bimodal breathing fish. It can breathe air at the water's surface when water oxygen concentrations are reduced (Ishimatsu and Itazawa, 1981) through accessory air-breathing with a suprabranchial organ (Ishimatsu and Itazawa, 1981; Graham, 1997; Lefevre et al., 2014). It has been reported that approximately 60% of the routine metabolism of the snakehead can be attributed to breathing air at the surface (Itazawa and Ishimatsu, 1981). This species is widely distributed in China. It is found throughout a broad temperature range of 0-30°C (Liu et al., 1998) and has recently become an invasive aquatic species in Europe and North America (Courtenay and Williams, 2004; Lapointe et al., 2013). It has been reported that the snakehead can move overland to more comfortable aquatic habitats (Courtenay and Williams, 2004) and may survive in air for several days within a temperature range of 10-15°C (Nagata and Nakata, 1988). As an invasive species, the terrestrial survival of the snakehead under extreme conditions may be of important ecological relevance. It could be assumed that the terrestrial survival of the snakehead is related to aerial respiratory capacity. However, no experimental data on the exact survival time of the snakehead in air has been documented to date. Therefore, the authors were interested in exploring how aerial respiration contributes to the survival of the snakehead in air.

The metabolic demands of fish may be enhanced by factors, such as higher temperatures (Jobling, 1994), feeding activities (Jobling, 1981) and intense exercise (Brett, 1972) which may cause an imbalance between oxygen supply and demand in extreme situations. It has been reported that the oxygen consumption rate (MO_2_) of the snakehead in water increases markedly when the temperature increases by Q_10_ values ranging from 1.31 to 4.00 (Xie et al., 2017) and increases after both feeding and exhaustive exercise (Wang et al., 2012; He et al., 2015). When the fish is out of the water, the imbalance between oxygen supply and demand may be more significant. However, it remains unclear whether the excessive metabolic demands of the snakehead can be fulfilled by its air-breathing capacity.

Metabolic suppression is a typical survival strategy for fish facing an adverse oxygen shortage (Smith, 1930; Storey and Storey, 1990). However, this may not hold true for fish that breathe air. It has been reported that for *Rivulus marmoratus* (Abel et al., 1987) and the Atlantic eel *Anguilla vulgaris* (Berg and Steen, 1965) the MO_2_ in water (MO_2Water_) is lower than the MO_2_ in air (MO_2Air_), indicating that these species use metabolic suppression strategies for aerial survival. In contrast, the MO_2Water_ of silver mudskipper *Periophthalmus sobrinus* (Gordon et al., 1969) and sculpin *Clinocottus analis* (Martin, 1991; Frick and Wright, 2002) did not differ from their MO_2Air_. Whether the snakehead adopts metabolic suppression when out of the water needs further study.

The objective of this study was to examine how the MO_2_ of the snakehead changes when it shifts from breathing water to breathing air and how the increased metabolic demand caused by temperature, feeding and exhaustive exercise affects its survival in air.

## RESULTS

**Fig. 1.**
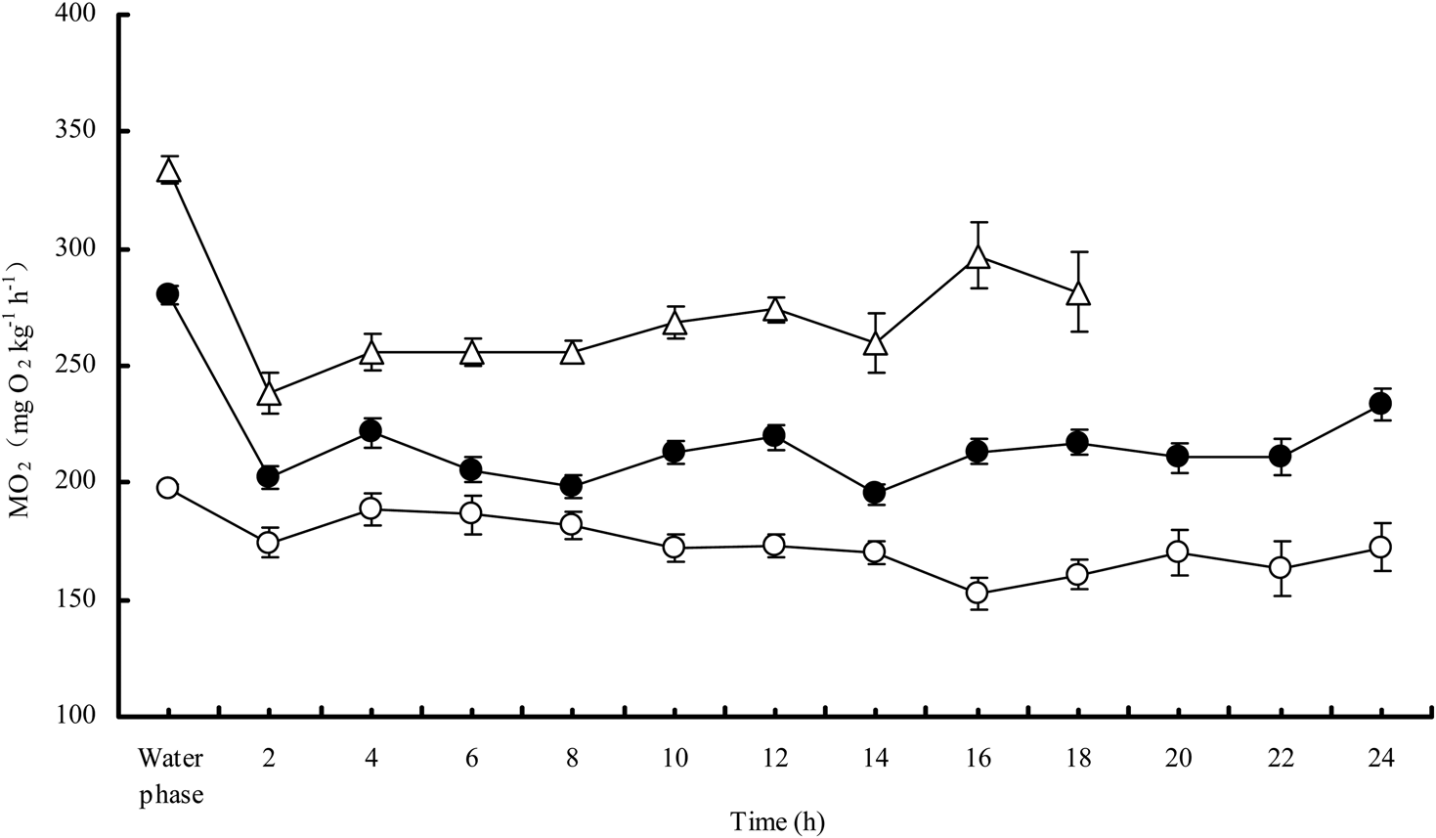
**Oxygen consumption rate (MO_2_) of the snakehead in water and in air at different temperatures.** The sample sizes were 14, 14 and 12 for 20, 25 and 30°C, respectively. Data are presented as mean±s.e.m. Open circles, 20°C; filled circles, 25°C; open triangles, 30°C.

**Fig. 2.**
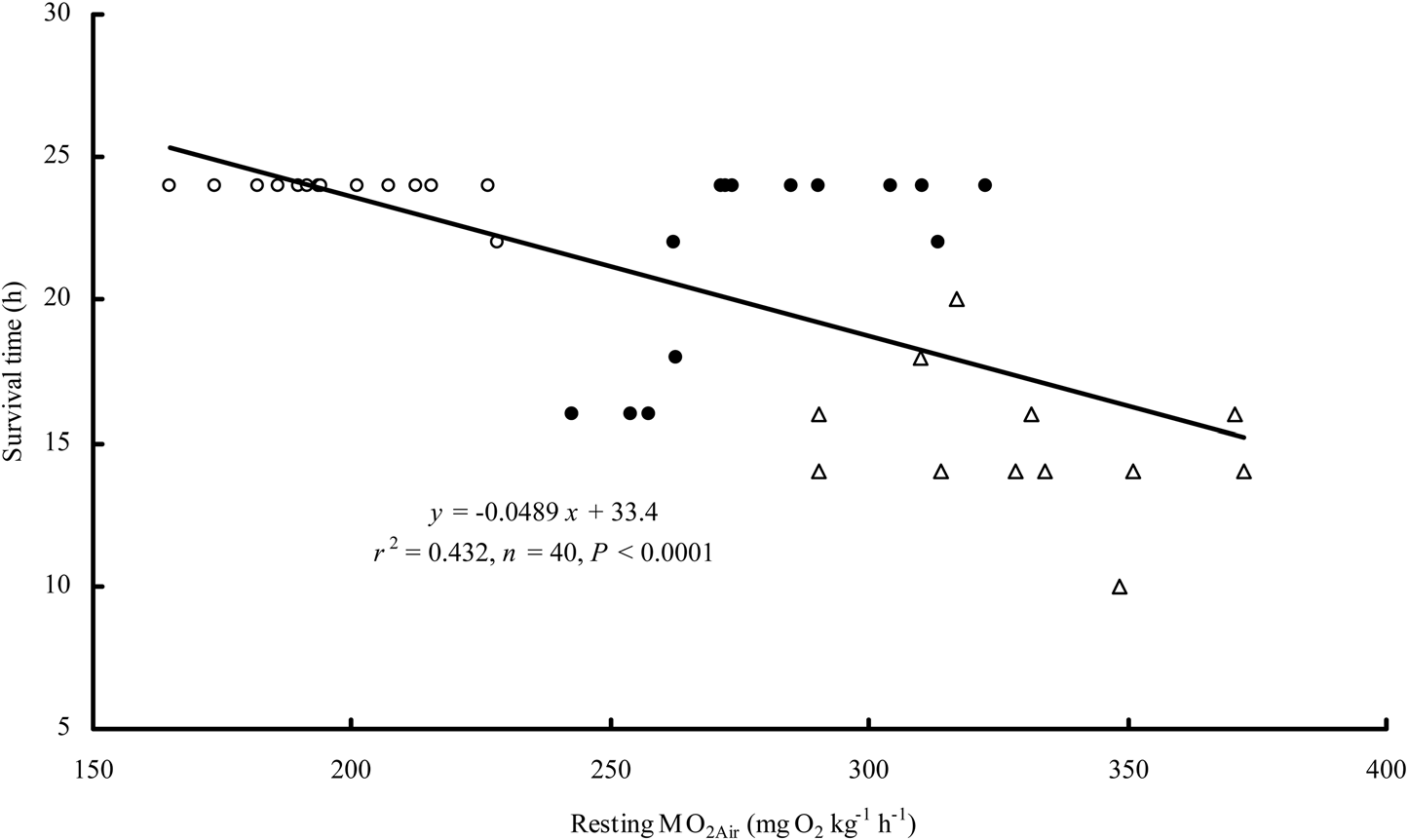
**The relationship between survival time and resting oxygen consumption rate in air (MO_2Air_) of the snakehead.** The correlation was significant (Pearson's correlation, *r*^2^=0.432, *n*=40, *P*<0.0001). The sample sizes were 14, 14, and 12 for 20, 25 and 30°C, respectively. Open circles, 20°C; filled circles, 25°C; open triangles, 30°C.

After moving from the water phase to the gas phase, the MO_2_ of the snakehead fell by 12.1, 24.5 and 20.4% at 20, 25, and 30°C, respectively. The resting MO_2Air_ was significantly lower than the resting MO_2Water_ at each temperature (*P*<0.0001). Both the resting MO_2Water_ and the resting MO_2Air_ increased with increasing temperature (Fig. 1). Q_10Water_ tended to decrease as temperature increased, while Q_10Air_ did not decrease (Table 1). The survival time was significantly shortened with increasing temperature (*P*<0.0001) (Table 1), and survival time was negatively correlated with the resting MO_2Air_ (*n*=40, *r^2^*=0.0432, *P*<0.0001) (Fig. 2).

**Table 1.**
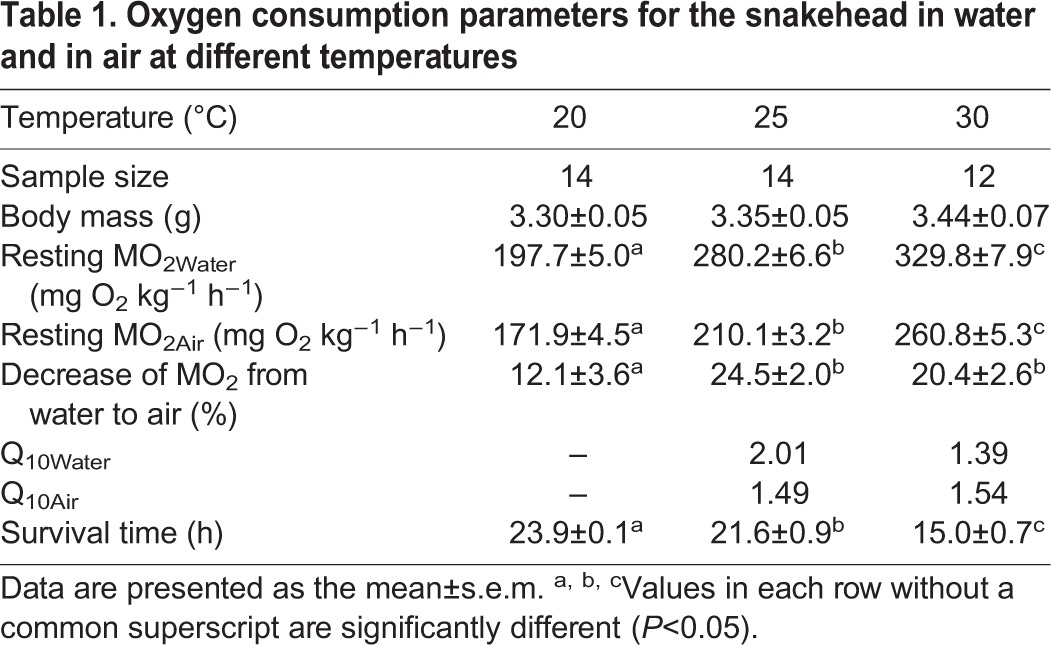
**Oxygen consumption parameters for the snakehead in water and in air at different temperatures**

**Fig. 3.**
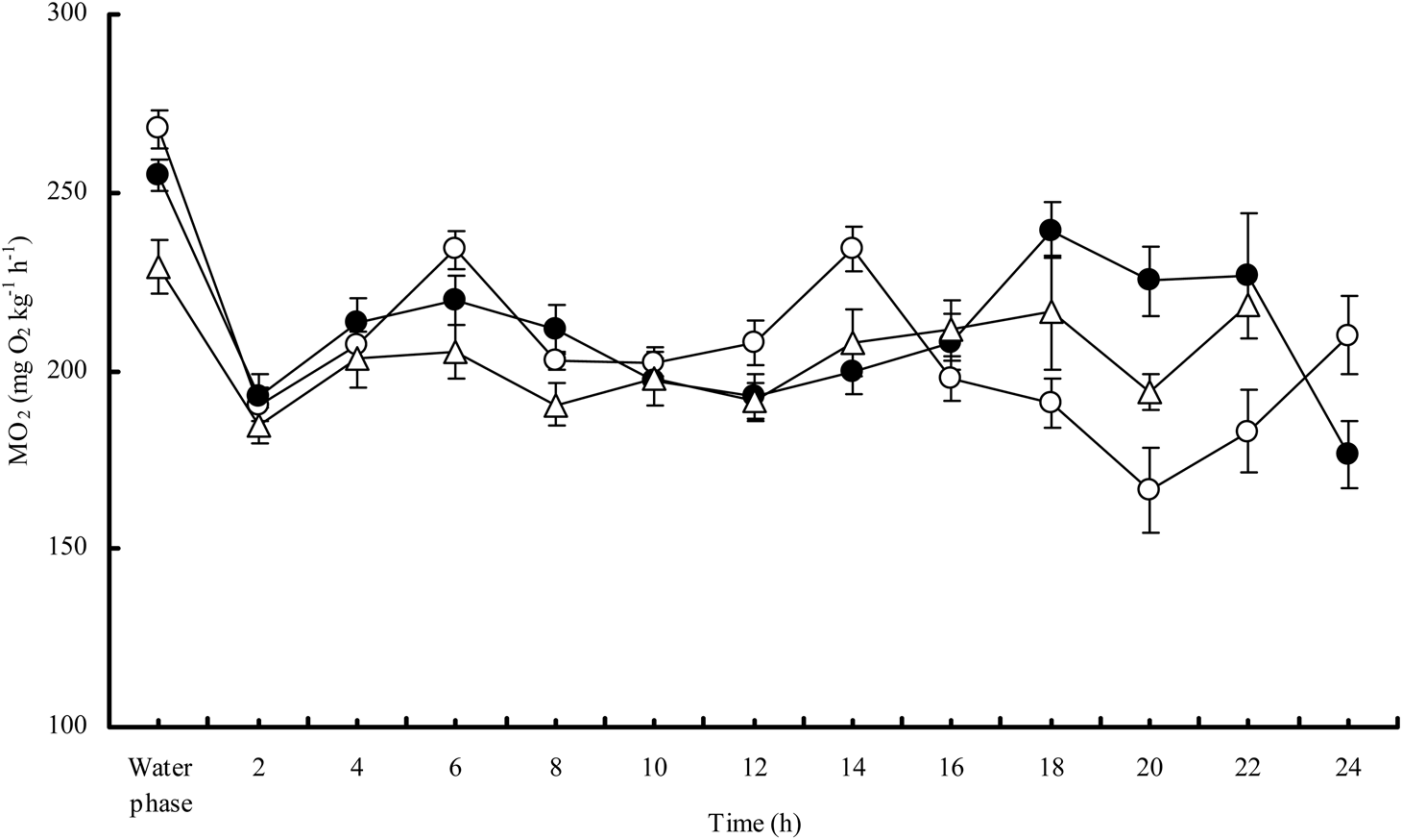
**Oxygen consumption rate (MO_2_) of the snakehead post-feeding in water and in air at 25°C.** The sample sizes were 14, 12 and 13 for 0, 1 and 3% feeding levels. Data are presented as mean±s.e.m. Open circles, fed at 0% body mass; filled circles, fed at 1% body mass; open triangles, fed at 3% body mass.

No obvious metabolic response was observed in the air-breathing snakeheads based on feeding behavior. The post-feeding MO_2Air_ of the snakehead was lower than the resting MO_2Water_ (Fig. 3), and no significant difference was observed among the three feeding levels (Table 2). The survival times of the snakehead fed at the 1% and 3% body mass levels were shorter than that of the control (*P=*0.018) (Table 2). The dry matter digestion rate was 57.6% for the snakehead fed at 1% and was significantly higher (30.3%) than the digestion rate of the fish fed at 3% body mass (*P=*0.004) (Table 2).

**Table 2.**
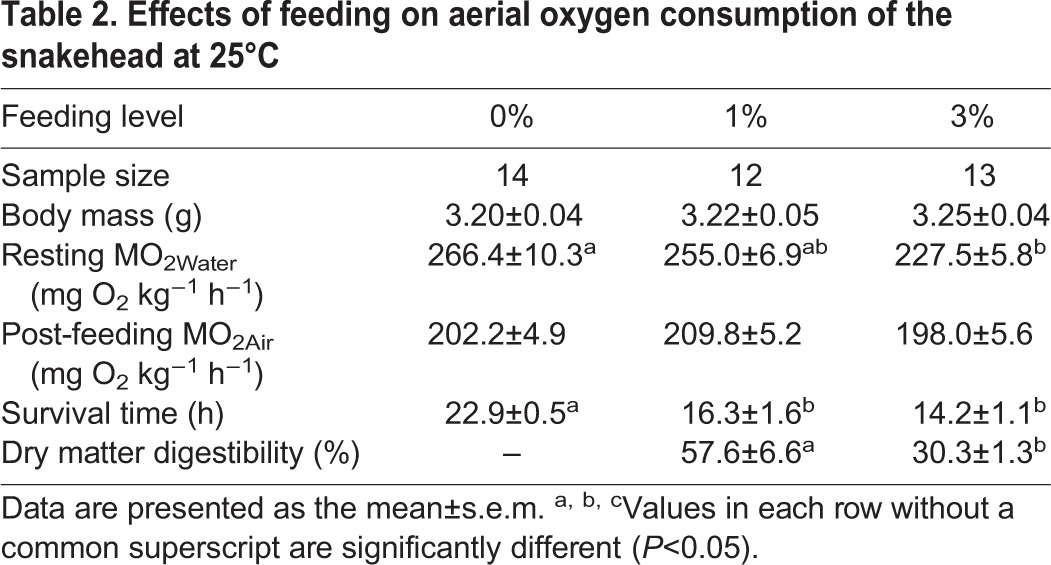
**Effects of feeding on aerial oxygen consumption of the snakehead at 25°C**

**Fig. 4.**
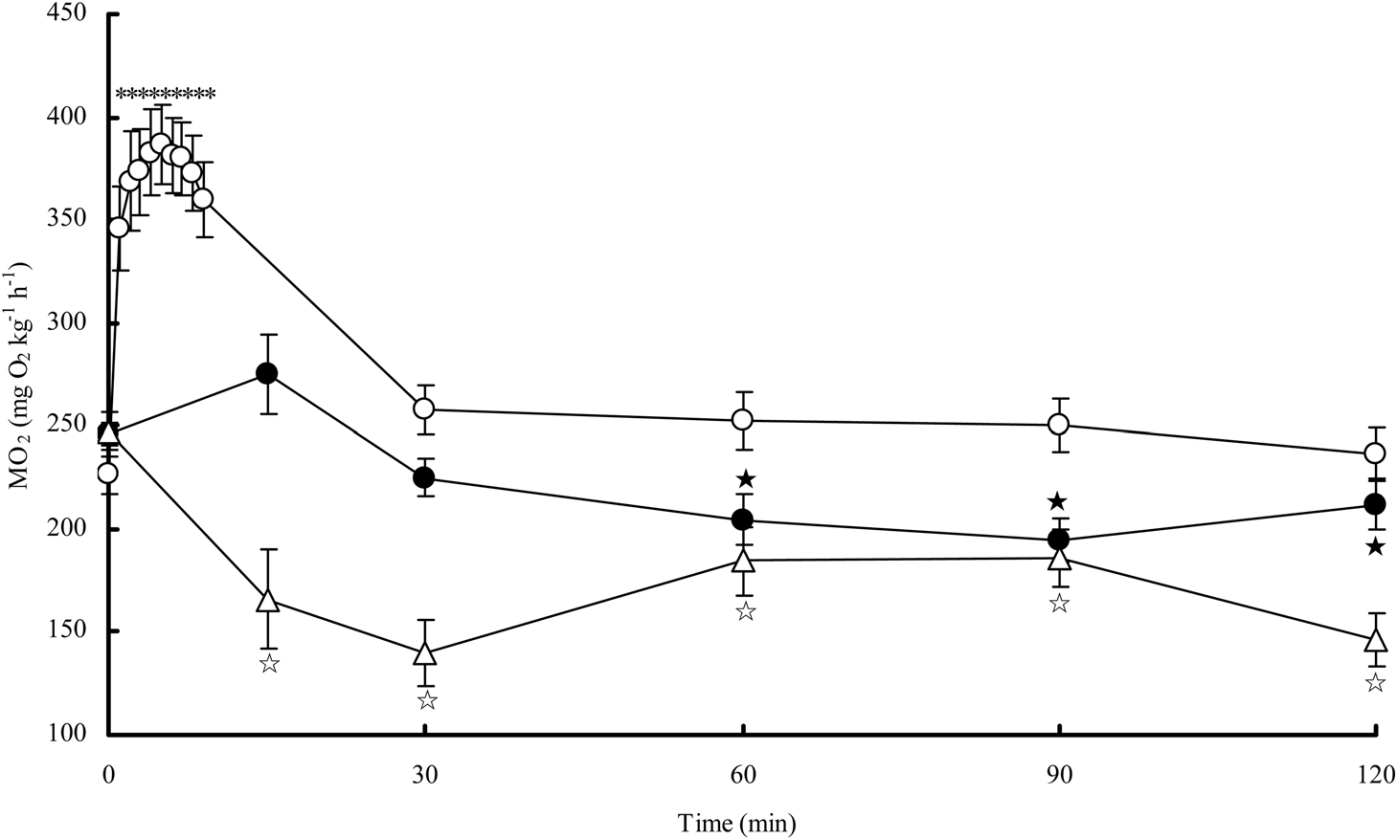
**Changes of aerial oxygen consumption of the snakehead post exhaustive exercise at 25°C.** The sample sizes were 12 for the water recovery group, 13 for the air recovery group, and 9 for the control group. Data are presented as mean±s.e.m. The values with symbols were different with pre-exercise levels of the fish recovery in water (asterisks), recovery in air (filled pentacles), and resting in air (open pentacles) by *t*-test, respectively (*P*<0.05).

**Fig. 5.**
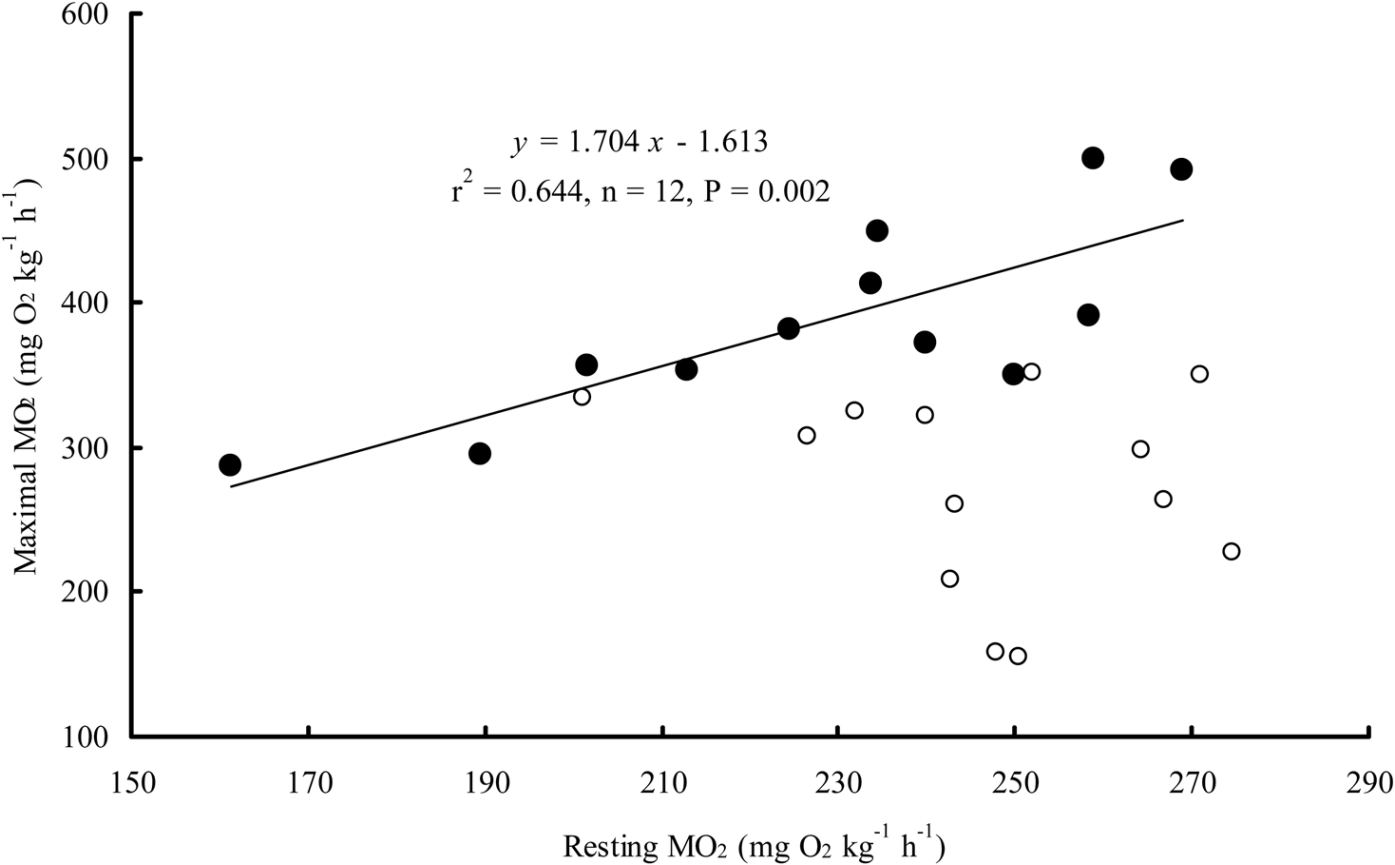
**The relationship between resting and maximal oxygen consumption rate (MO_2_) of the snakehead recovery in water and air.** Open circles, the fish recovery in air; filled circles, the fish recovery in water. The correlation for the fish recovery in water was significant (*r*^2^=0.644, *n*=12, *P*=0.002), but not for the fish recovery in air (Pearson's correlation, *r*^2^=0.0471, *n*=13, *P*=0.225).

The MO_2Water_ of the snakeheads after exhaustive exercise tended to increase to a peak value higher than the resting MO_2Water_ (*P<*0.0001). However, only a slight increase in MO_2Air_ was observed in the snakehead after exhaustive exercise (Fig. 4). The resting MO_2Water_ was positively correlated with the maximum MO_2Water_ of the snakehead after exhaustive exercise (*n*=12, *r^2^*=0.644, *P=*0.002) (Fig. 5) but was not correlated with the maximal MO_2Air_ after exhaustive exercise. The maximum MO_2Air_ of the snakehead after exhaustive exercise was significantly higher than the MO_2Air_ in the control group (*P=*0.001). Recovery time for MO_2Water_ was 98 min, which was significantly longer than the recovery time for MO_2Air_ (*P<*0.0001) (Table 3).

**Table 3.**
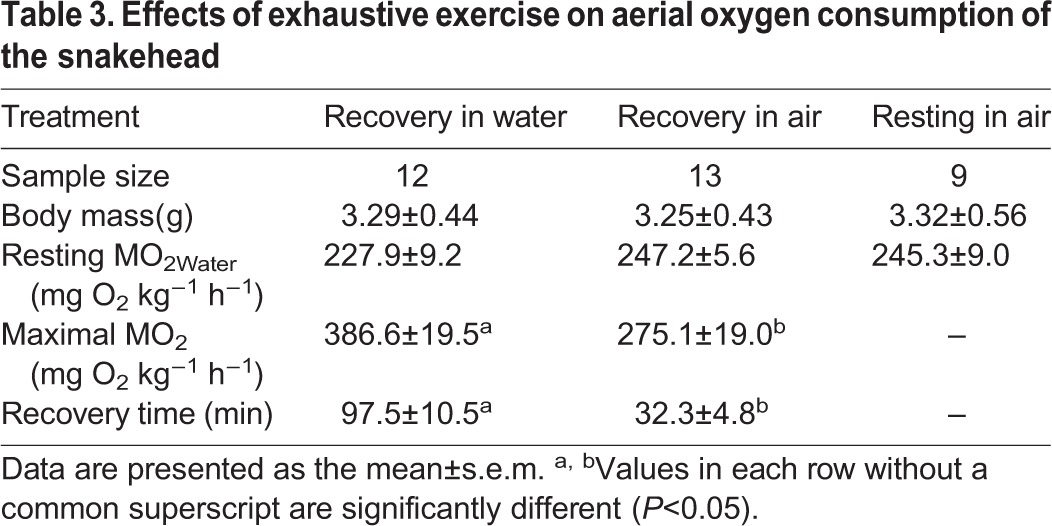
**Effects of exhaustive exercise on aerial oxygen consumption of the snakehead**

## DISCUSSION

Our results showed that the snakehead could survive out of water by breathing air for varying lengths of time depending on ambient temperature and metabolic demand. The resting MO_2Water_ was close to that previously reported for the snakehead at the same temperature in water (Wang et al., 2012; Xie et al., 2017). The resting MO_2Water_ increased with increasing temperature, consistent with the previous studies on this species (Liu et al., 2000), and with the general metabolic response of fish to temperature change (Jobling, 1981; Luo and Xie, 2008, 2009). Q_10Water_ decreased at higher temperatures (Table 1), suggesting a slower increase in metabolism at higher temperatures. This pattern has been well documented and shown to be the result of higher metabolic demand and lower oxygen availability in water at higher temperatures. Similarly, the snakeheads' resting MO_2Air_ also increased with increasing temperature (Fig. 1), which suggests that the capacity of the snakehead to breathe air is enhanced to some extent at higher temperatures. However, one interesting result of our study was that the Q_10Air_ of the snakehead did not decrease as temperature increased (Table 1), indicating no significant limitation of oxygen availability in air at higher temperatures. This could be explained by the fact that oxygen content and diffusivity in air is higher than in water and can be sustained with shifts in temperature (Fusi et al., 2016). Therefore, the shorter survival time of the snakehead in the air at warmer temperatures may not be primarily due to the imbalance of oxygen supply and demand. Other factors may affect its aerial survival, such as an increase in endogenous ammonia content (Gordon et al., 1969; Chew et al., 2003), and uncompensated respiratory acidosis by metabolically produced CO_2_ accumulation (Ishimatsu and Itazawa, 1981, 1983) which has deleterious effects on the bodies of fish (Ip et al., 2001; Walsh, 1998).

The resting MO_2_ of the snakehead decreased as it shifted from breathing water to air regardless of temperature, suggesting that metabolic depression is occurring to some extent. This could be a strategy to reduce metabolic demand and prolong survival in air. In fact, survival time when breathing air was negatively correlated with resting MO_2Air_ (Fig. 2), demonstrating the remarkable survival advantages of metabolic depression for the snakehead. Alternatively, another possible explanation for the lower MO_2_ in air could be the reduced cost of ventilation in air for the greater oxygen availability and the lower viscosity compared to in water. The decrease in resting MO_2_ of the snakehead after moving from water to air was smaller at 20°C than at higher temperatures (Table 1). This could be due to its lower metabolic demand at lower temperatures and suggests that the snakehead's accessorial respiration contributes more to its aerobic metabolism in cold environments.

An apparent feeding metabolic response has been reported in the snakehead in water fed with the same meal size as in the present study (Wang et al., 2012). A meal size of 1 to 3% can induce a factorial metabolic scope of 1.68 to 1.84 and the feeding metabolic response can last for 14.8 to 23.0 h (Wang et al., 2012). However, our results showed neither an apparent feeding metabolic response in the snakehead when in air (Fig. 3) nor any significant differences between feeding levels in the post-feeding MO_2Air_ (Table 2). This suggests that the snakehead's accessorial respiration can sustain only the basal metabolic demand but cannot meet the extra demand of the feeding metabolism. It has been proposed that a small feeding metabolic response may also be related to weak food digestion (Secor, 2009). Indeed, only a small amount of food was digested by the snakehead in our study (Table 2), which has contributed to its limited postprandial metabolic response when breathing air.

Previous studies have observed that the MO_2Water_ of fish after exhaustive exercise generally increases and reaches its peak rapidly (Hicks and Bennett, 2004; Fu et al., 2007; Wang et al., 2012), and this was also observed in our study (Fig. 4). The MO_2Air_ of the snakehead after exhaustive exercise was not notably higher than the pre-exercise level but was higher than the MO_2Air_ of the control group, suggesting that the metabolic capacity of the snakehead can still up-regulate to some extent when it is facing excess metabolic demand for activity while in air. In this study, individuals with a higher resting MO_2Water_ had a higher maximum MO_2Water_ after exercise, but the maximum MO_2Air_ after exercise was not necessarily higher (Fig. 5). This indicates that the aerobic capacity of the snakehead may reach its upper limit when breathing air, apparently narrowing the differences among individuals.

In conclusion, the juvenile snakeheads could survive out of water by breathing air for 15-24 h within the temperature range of 20-30°C. Additionally, some degree of metabolic depression occurs in the snakehead when in air. The metabolic demand associated with exercise of the snakehead but not that associated with feeding can be supported by the fish's air-breathing capacity to some extent.

## MATERIALS AND METHODS

### Experimental animals

Juvenile snakeheads were obtained from the Huashan hatchery in Guangdong, China and were held in a rearing system for 2 weeks prior to experiment. The fish were fed to satiation twice daily (11:30 and 18:30) with cutlets of loach *Misgurnus anguillicaudatus* with viscera, head and tail removed. During acclimation, the photoperiod was 12 h light:12 h dark and the water temperature was 25.0±1°C. One third of the water was refreshed with aerated water each day, and the dissolved oxygen was kept above 7 mg O_2_ l^−1^ using a continuous aeration The ammonia-nitrogen concentration was kept below 0.015 mg l^–1^. Animals were handled according to the ethical requirements for animal care of the Fisheries Science Institution of Southwest University of China, and the study followed the required standards for environmental and housing facilities for laboratory animals in China (Gb/T14925-2001).

### Measurement of MO_2_

MO_2Water_ and MO_2Air_ were determined using a flow-through respirometer consisting of multiple plexiglass chambers immersed in a temperature-controlled water bath. The chamber (30 ml) was composed of connecting inlet and outlet triple valves and could be easily switched from a flow-through water phase to a closed air phase. The dissolved oxygen concentration was measured at the outlet by using a fiber optic sensor system (Mircrox TX3, PresSen - Precision Sensing GmbH Regensburg, Germany). Before data was collected, the water flow rate was adjusted to ensure that the dissolved oxygen in the outlet water was approximately 1 mg O_2_ l^−1^ lower than that of the control while maintaining a saturation concentration higher than 70% to avoid hypoxia stress (Blaikie and Kerr, 1996; Fu and Xie, 2004). Velocity was measured by collecting water over a set period of time. To reduce the influence of circadian rhythm, a full-light (24 h light:0 h dark) environment was used during MO_2_ measurement (Wang et al., 2012). The following formula was used to calculate MO_2Water_ (mg O_2_ kg^−1^ h^−1^): MO_2Water_=ΔDO_2_×v/m, where ΔDO_2_ (mg O_2_ l^−1^) is the difference between the dissolved oxygen concentration in the fish chamber and the control chamber, v (l h^−1^) is the water flow rate, and m (kg) is the wet mass of the fish. After determining MO_2Water_, the inlet was switched from the water pump to the air pump, and the water in the chamber was discharged completely for MO_2Air_ measurement. Approximately 0.4 ml of water was added to the chamber to maintain near-saturated humidity conditions in the chamber, and the fish were acclimated for 30 min. MO_2Air_ was determined in an intermittent flow pattern. A 1-ml syringe was connected to the inlet valve to determine the change in air volume due to air breathing. The initial oxygen partial pressure of the air was measured using an oxygen probe, and the chamber was sealed for 90 min. Then, the final oxygen level of the air was measured. In this study, the level of saturated oxygen partial pressure was maintained above 70%. The inlet and outlet were opened immediately, and the air in the chamber was refreshed for 30 min. Then, the next determination loop was initiated. Meanwhile, atmospheric pressure was recorded during each measurement. Oxygen concentration in the air (mg O_2_ l^−1^) was obtained from the values of oxygen partial pressure, temperature, and atmospheric pressure. MO_2Air_ (mg O_2_ kg^− 1^ h^− 1^) was calculated as: MO_2Air_=(O_2i_×V_i_−O_2f_×V_f_)/t/m, where O_2i_ (mg O_2_ l^−1^) is the initial oxygen concentration of the air, V_i_ (l) is the initial air volume, O_2f_ (mg O_2_ l^−1^) is the final oxygen concentration of the air, V_f_ (l) is the final air volume, t (h) is the breathing time, and m (kg) is the wet mass of the fish. The volume of the fish body was calculated assuming a body density of 1 kg l^−1^. One chamber without fish was set as the control chamber.

### Experimental processes

#### Effect of temperature

The three test temperatures used were 20, 25 and 30°C, and variations in these temperatures were less than 0.2°C. At each temperature, 14 snakehead individuals (body mass approx. 3 g) were treated for 2 weeks. Other than temperature, housing conditions were the same as in the previous experimental period. At the end of temperature treatment, the fish were weighed individually after fasting for 24 h and were placed into the respiratory chamber for an additional 24 h. The MO_2Water_ was measured at 1 h intervals for 8 h, and the average value was taken as the resting MO_2Water_ for that individual. Then, the respiratory chamber was switched to the air phase for MO_2Air_ measurement. The MO_2Air_ was measured at 2 h intervals for 24 h or until the fish died and was averaged to calculate the resting MO_2Air_ for each individual. The survival duration of each individual was recorded during this process. The final numbers of fish tested using this process were 14, 14 and 12 for 20, 25 and 30°C, respectively. The temperature quotient (Q_10_) was calculated as: Q_10_=(MO_2a_/MO_2b_)^10/(Ta-Tb)^, where MO_2a_ and MO_2b_ are the average metabolic rates at temperatures T_a_ and T_b_, respectively.

#### Effects of feeding

After fasting for 24 h, the fish were weighed and placed into the respiratory chamber for 24 h of acclimation. The water temperature was kept at 25.0±0.2°C. The three feeding levels tested were 0% (control group), 1% and 3% of body mass, since the same post-feeding maximum metabolic rate was observed in the snakehead fed at 3% body mass and at the largest level of 5% body mass (Wang et al., 2012). After determining resting MO_2Water_, the snakeheads were fed with loach cutlets using a gavage protocol as described by Li et al. (2010). MO_2Air_ was then measured at 2 h intervals for 24 h or until the fish died. The averaged value was recorded as the post-feeding MO_2Air_. The final numbers of fish tested using this process were 14, 12 and 13 for 0%, 1% and 3% feeding levels, respectively. After measuring MO_2Air_, the fed fish was removed from chamber and undigested cutlets were removed through intestinal dissection. The undigested cutlets were weighed and dried at 70°C. The dry matter digestion ratio (DR) was calculated as: DR(%)=[(I−E)/I]×100, where I (g) is the dry mass of food intake and E (g) is the dry mass of undigested food.

#### Effects of exhaustive exercise

The fish were divided into three groups: water recovery, air recovery, and resting in air (control group). After 24 h of fasting, healthy juvenile snakeheads were selected randomly and weighed (approx. 3 g). Then, individuals were placed into the respiratory chamber for 24 h of acclimation. The water temperature was maintained at 25.0±0.2°C. MO_2Water_ was measured at 1 h intervals for 8 h, and the average value was recorded as the resting MO_2Water_. Then, each fish was transferred from the chamber to a chasing channel (150 l), where it was chased constantly by hand and swam for 10 min until exhaustion. Fish were deemed exhausted after losing body balance or displaying no significant reaction to being chased (Fu et al., 2007). The exhausted fish were immediately transferred back into the respiratory chamber and the process of determining post-exercise MO_2Water_ or MO_2Air_ during recovery was initiated_._ For the water recovery group, MO_2Water_ was measured at 1 min intervals for 10 min according to the methods presented by Huang et al. (2013) and then measured again at 30, 60, 90 and 120 min after exercise. The peak value was taken as the maximum MO_2Water_ after exhaustive exercise. For the air recovery group, the exhausted fish was placed back into the respiratory chamber without water, and MO_2Air_ was measured at 15, 30, 60, 90 and 120 min after exercise. The control group did not undergo exhaustive exercise and MO_2Air_ was determined after MO_2Water_ was determined. The final numbers of fish tested using this process were 12 for the water recovery group, 13 for the air recovery group, and 9 for the control group.

### Statistical analysis

Experimental results were calculated using Microsoft Excel 2003 (Microsoft Corporation, Redmond, WA, USA), and statistical analyses were performed using SPSS 11.5 (SPSS Inc., Chicago, IL, USA). Parameters among groups were compared using one-way ANOVA followed by an LSD test. A *t*-test was used to compare within group values measured before and after feeding, and before and after exercise. An ordinary least squares (OLS) regression was used to analyze the relationship between MO_2_ values and survival time. The relationship between resting MO_2_ and maximum MO_2_ was analyzed using Pearson's correlation. Differences were considered significant when the *P*<0.05. Data are presented as the mean±s.e.m.
